# Tall Cell Carcinoma with Reverse Polarity of Breast: Report of a Case with Unique Morphologic and Molecular Features

**DOI:** 10.5146/tjpath.2020.01511

**Published:** 2021-05-15

**Authors:** Mohamed Jassim, Chennagiri S Premalata, Geeta V Patil Okaly, Chunduri Srinivas

**Affiliations:** Department of Pathology, Kidwai Memorial Institute of Oncology, Bengaluru, India; Department of Surgical Oncology, Kidwai Memorial Institute of Oncology, Bengaluru, India

**Keywords:** Breast carcinoma, Tall cell carcinoma, Reverse polarity, Solid papillary, IDH2

## Abstract

Breast carcinomas are a heterogeneous group of malignancy, having variable clinical outcomes depending on their cellular and molecular properties. Tall cell carcinoma with reverse polarity (TCCRP) is a recently described rare entity, which shares morphological features with tall cell variant of papillary thyroid carcinoma but has a distinct morphological, immunohistochemical, and molecular profile. We describe a case of a 40-year-old female patient, who presented with lump in the breast. The patient underwent lumpectomy and was diagnosed as tall cell carcinoma with reverse polarity. Immunohistochemistry and bi-directional Sanger sequencing for IDH2 mutation were used for diagnosis. Tall cell carcinoma with reverse polarity is a rare and newly described entity with characteristic morphological and molecular findings, which carries an excellent prognosis.

## INTRODUCTION

Tall cell carcinoma with reverse polarity (TCCRP) is a rare type of invasive breast carcinoma with characteristic immuno-morphologic and molecular findings and is often difficult to diagnose. Eusebi et al., in 2003, reported a rare variety of breast carcinoma having features similar to papillary thyroid carcinoma described as “Breast tumor resembling the tall cell variant of papillary thyroid carcinoma” (1). These tumors were also called breast tumor resembling the tall cell variant of papillary thyroid carcinoma; solid papillary breast carcinoma resembling tall cell variant of papillary thyroid carcinoma; solid papillary carcinoma with reverse polarity (1–5). These tumors were consistently immuno-negative for TTF-1, thyroglobulin and HBME1, and there was no associated thyroid malignancy. Chiang et al. described 13 more cases and also identified the IDH2 hotspot mutations at R172 in these tumors, which has become a defining feature of this entity (4). We are presenting a case report of this rare entity in a 40-year-old female, currently named “Tall cell carcinoma with reverse polarity” by the World Health Organization (6) and there are only scattered case reports and very few case series published in the world literature to the best of our knowledge.

## CASE REPORT

**Figure 1:**
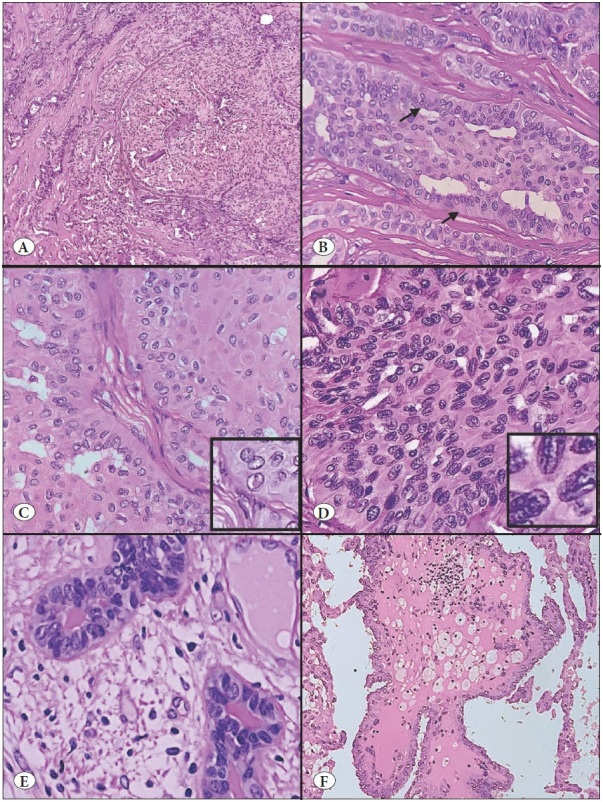
Histomorphological features of Tall cell carcinoma with reverse polarity (TCCRP). **A)** Tumor composed of compact solid nests in a background of fibrotic stroma, (H&E; x40). **B)** Nest and papillae are lined by tall columnar epithelial cells, nuclei exhibiting reverse polarity (arrow) (H&E; x100). **C)** Tumor cells have eosinophilic granular cytoplasm, oval nuclei with chromatin clearing, and intranuclear pseudoinclusion (Inset show intranuclear pseudo inclusion) (H&E; x100). **D)** Nuclei exhibit crowding, overlapping and nuclear groove (Inset show nuclear groove) (H&E; x400). **E)** Follicular structures with eosinophilic/amphophilic colloid-like secretions (H&E; x100). **F)** Fibrovascular core with foamy macrophages (H&E x100).

A 40-year-old female presented with pain and a palpable lump in the right breast for the past one month. Past history and family history were not significant. Mammography revealed a BIRADS IV lesion in the upper outer quadrant. Fine needle aspiration cytology was reported as proliferative breast disease with atypia, and trucut biopsy was inconclusive for malignancy. Hence patient underwent lumpectomy, which was followed later by modified radical mastectomy with axillary clearance. The nodular lesion excised was relatively circumscribed, grey-white to yellow in color, firm in consistency, and measuring 5.5x4.5x4cm. The resection margin was formed by the lesion. Grossly, no areas of necrosis/hemorrhage were identified. Microscopy showed a tumor composed of nodules of neoplastic cells arranged in nests and solid papillary patterns with thin fibrovascular cores. Papillae were lined by tall columnar epithelial cells, having a moderate amount of granular eosinophilic cytoplasm, oval nuclei with moderate nuclear pleomorphism, nuclear overlapping, crowding, nuclear grooves, optical clearing of chromatin, and occasional intranuclear pseudo-inclusions. The linear arrangement of nuclei situated away from the basement membrane, so-called reverse polarity, was also seen at places. Foci with fibrovascular cores having clusters of foamy macrophages and follicular structures with colloid-like eosinophilic secretion were also noted. Mitosis was sparse (1-2 per 10 high power field). No necrosis, lymphovascular emboli, or perineural invasion were noted (Figure 1).

**Figure 2:**
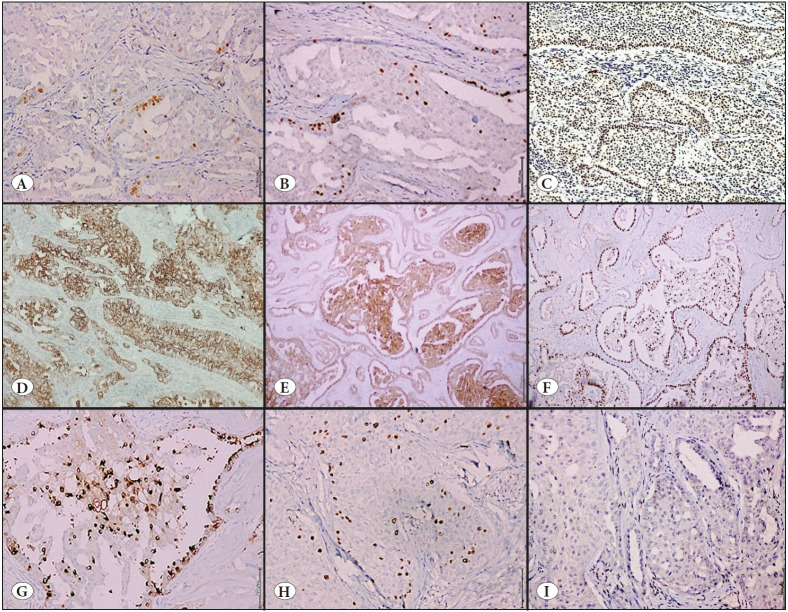
**A)** Neoplastic cells show weak and patchy expression for ER (IHC; x100) and **B)** PgR (IHC; x400). **C)** AR showed heterogenous staining pattern with intense staining at the peripheral of the tumor (IHC; x100). **D)** Neoplastic cells are positive for CK5/6 (IHC; x40), **E)** Calretinin (IHC; x40), **F)** GATA-3 (IHC; x40), **G)** with luminal expression of EMA (IHC; x400). **H)** Low Ki67 proliferation (IHC; x100) and **I)** negative for TTF-1 (IHC; x40).

Immunohistochemistry was performed using an automated stainer (Ventana Benchmark XT system®) with Ultra-View Universal DAB detection. The antibodies used were GATA3, GCDFP-15, estrogen receptor (ER), progesterone receptor (PgR), Androgen receptor (AR), HER2/neu, synaptophysin, chromogranin A, Bcl2, EMA, CK 5/6, p63, thyroid transcription factor 1 (TTF-1), inhibin, and Ki67. Antibody details are shown in Table 1. The neoplastic cells were immuno-positive for GATA3, CK5/6, EMA and calretinin with patchy and weak expression for ER and PgR. AR showed heterogenous staining pattern with peripheral accentuation in the tumor lobules (Figure 2), which has not been described earlier in the literature. While neoplastic cells were immuno-negative for HER2/neu, synaptophysin, chromogranin, Bcl2, GCDFP-15, TTF1, and inhibin, the Ki67 proliferation index was 5%. No staining for p63 was seen around the lobules. Immunohistochemical features of the tumors are illustrated in Figure 2.

**Table 1:** Details of primary antibodies.

**Anti-sera**	**Source**	**Clone**	**Dilution**
**AR**	BioGenex	F39.4.1	1:20
**Bcl-2**	Bio SB	BSB-5	1:200
**Calretinin**	BioGenex	2E7	1:30
**CK5/6**	Bio SB	B5-16B4	1:150
**Chromogranin**	Bio SB	LK2H10	1:4000
**EMA**	BioGenex	E29	1:120
**ER**	Roche	SP1	Pre-diluted
**GATA3**	Bio SB	L50-823	1:100
**GCDFP-15**	BioGenex	EP95	1:50
**HER 2**	Roche	4B5	Pre-diluted
**Inhibin**	BioGenex	R1	1:30
**Ki67**	BioGenex	MIB-1	1:50
**PgR**	Roche	1E2	Pre-diluted
**P63**	BioGenex	4A4	1:50
**Synaptophysin**	BioGenex	SMP 88	1:150
**TTF-1**	BioGenex	SP141	1:80

**AR:** Androgen receptor, **CK:** Cytokeratin, **EMA:** Epithelial membrane antigen, **ER:** Estrogen receptor, **GATA3:** GATA binding protein-3, **GCDFP-15:** Gross cystic disease fluid protein 15, **HER2:** human epidermal growth factor 2, **PgR:** progesterone receptor, **TTF-1:** Thyroid transcription factor 1.

**Figure 3:**
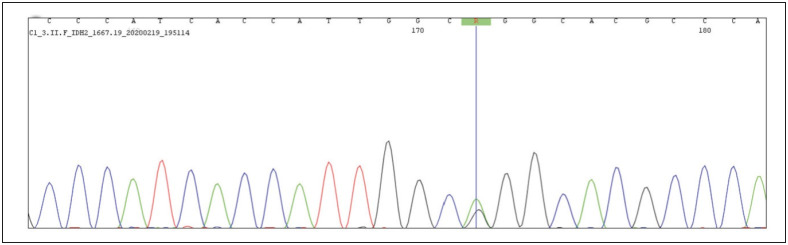
DNA sequence chromatogram showing mutation in IDH2 gene at R172.

DNA was isolated from formalin-fixed paraffin-embedded blocks and targeted sequencing for the commonly mutated site of IDH1 (R132) and IDH2 (R172) was performed through a bi-directional Sanger sequencing method on the amplified template. The tumor tissue showed a mutation in the IDH2 gene (DNA description: c.A514>G; Protein description: p.Arg172Gly – R172G). DNA sequence chromatogram for the IDH2 gene is shown in Figure 3. Based on the characteristic immuno-morphology and molecular findings, a diagnosis of tall cell carcinoma with reverse polarity was made. Following lumpectomy, completion mastectomy with axillary lymph node dissection was performed owing to the positive surgical margin. Grossly and microscopically, no residual tumor was identified and all nine axillary lymph nodes were free of tumor. PET-CT scan revealed no evidence of metastasis. No further treatment was given owing to the indolent behavior of the tumor, and the patient is under observation and is currently disease-free for 6 months post-surgery. Written informed consent for publication was taken from the patient.

## DISCUSSION

Tall cell carcinoma with reverse polarity is a recently described invasive breast carcinoma, included in the 5th edition of WHO classification of breast tumors as a slow-growing tumor with favorable prognosis (1–6). These tumors most commonly occur in women around the age of 65 years (range 52 to 75years), with characteristic immuno-morphologic features. Molecular studies have shown IDH2 mutation in almost all of these tumors, which has become a defining feature. Morphologically, they form well-circumscribed masses with cells arranged in compact solid nests in a background of fibrotic stroma, having thin fibrovascular cores that recapitulates a solid-papillary pattern, and sometimes the cores contain foamy macrophages. Papillae are lined by tall columnar cells having abundant eosinophilic cytoplasm, bland oval to round nuclei exhibiting nuclear grooves and intranuclear cytoplasmic inclusions (1,3). The most characteristic feature is apical location of the nuclei resulting in reverse polarity. Other features include low mitotic count and absent myoepithelial cells at the periphery and within the papillary fronds. Psammoma bodies, granular calcification, and follicular structures with eosinophilic/amphophilic colloid-like secretions are sometimes noted (7). Areas of necrosis, lymphovascular emboli, or peri-neural invasions are usually not seen (8). The present case showed all the above characteristic morphological features. These tumors show weak or absent ER, PgR, AR expression, and are almost always negative for HER2/neu. Although morphological features of TCCRP resemble papillary thyroid carcinoma, they are negative for TTF-1, thyroglobulin and HBME 1 (9,10), and the breast origin of the neoplasm is confirmed by the positive staining for markers of mammary differentiation like GATA3, mammaglobin and GCDFP-15 (7,8,10,11). Myoepithelial markers p63, SMMHC, and calponin are absent within and around the lesion, suggestive of invasive disease. Neoplastic cells are also immuno-positive for CK5/6, and focally for EMA, CEA, and bcl-2, and exhibit a low Ki67 proliferation index ranging from 1 to 5% (3,5,8). All TCCRP also show diffuse and strong expression calretinin. The immunohistochemical profile in the present case was similar to the cases described in the literature, with strong expression of GATA3, CK5/6, calretinin, and weak expression of hormone receptors, and AR showed a heterogenous staining pattern with peripheral accentuation in the tumor lobules (Figure 2), which has not been described earlier in the literature. Ki67 proliferation was low (5%). In addition, the neoplastic cells showed patchy expression of GCDFP15 and luminal expression of EMA (12). Chiang et al. performed molecular analysis on 13 cases of TCCRP using whole-exome and targeted sequencing and found that 77% of cases harbored IDH2 mutation at R172 (4). Alsadoun et al., after genetic and transcriptomic profiling of this tumor using whole-exome analysis, showed that the IDH2 gene harbors a hotspot mutation at the level of arginine 172 (R172), which was not present in any other breast carcinoma, in 78% of TCCRP (7). Although IDH2 mutations are commonly identified in many tumors such as secondary gliomas, myeloid malignancy, cholangiocarcinoma, microsatellite stable colorectal cancer, chondrosarcoma etc., they are infrequent among breast tumors but are typically seen in TCCRP (7,13–17). Pareja et al. demonstrated that immunohistochemistry using a monoclonal antibody against IDH2 R172S (Clone - 11C8B1) is sensitive and specific for TCCRP harboring the IDH2 R172 hotspot mutation (18). Genetic alterations prevalent in papillary thyroid carcinoma like RET/PTC rearrangements and BRAF mutations (18,19) are almost always absent in TCCRP. Bi-directional Sanger sequencing on the paraffin block showed IDH2 hotspot mutations at R172 in the present case, which is a characteristic and unique molecular finding of this tumor. TCCRP has an indolent clinical behavior with favorable outcomes with surgery being the mainstay of treatment (3,4,8,20). It has the potential for recurrence in case of incomplete excision of the tumor; thus complete excision with adequate margin is the optimal treatment for TCCRP (3). However, two instances of metastasis have been reported in literature: Cameselle-Teijeiro et al. reported one case with bone metastasis and Foschini et al. reported one case with intramammary lymph node metastasis (5,8).

TCCRP should be differentiated from other papillary lesions of the breast like encapsulated or solid papillary carcinoma and invasive micropapillary carcinoma as it shares papillary architecture with lack of myoepithelial cells. TCCRP can be differentiated from these entities by the characteristic morphology of cells with reverse polarity and immuno-positivity for CK5/6, calretinin and negative or weak expression of hormonal receptors. Intraductal papilloma with usual ductal hyperplasia shares nuclear groove and pseudo-inclusion with TCCRP but can be differentiated by the presence of myoepithelial cell layer in papilloma and usual ductal hyperplasia, while TCCRP lacks a layer of myoepithelial cells.

In conclusion, TCCRP is a rare and newly described entity with characteristic morphological findings that can mimic other papillary lesions of the breast. IDH2 hotspot mutation is a distinctive and characteristic finding in TCCRP. A high index of suspicion is necessary to avoid misdiagnosis of TCCRP as other papillary breast lesions or metastasis from the thyroid, and accurate diagnosis of this entity is necessary as it carries an excellent prognosis.

## Conflict of INTEREST

None of the authors have any competing interests.

## FUNDING

None

## References

[ref-1] Eusebi V., Damiani S., Ellis I. O., Azzopardi J. G., Rosai J. (2003). Breast tumor resembling the tall cell variant of papillary thyroid carcinoma: report of 5 cases. Am J Surg Pathol.

[ref-2] Tosi Anna L., Ragazzi Moira, Asioli Sofia, Del Vecchio Marina, Cavalieri Monica, Eusebi Leonardo H. U., Foschini Maria P. (2007). Breast tumor resembling the tall cell variant of papillary thyroid carcinoma: report of 4 cases with evidence of malignant potential. Int J Surg Pathol.

[ref-3] Bhargava Rohit, Florea Anca V., Pelmus Manuela, Jones Miroslawa W., Bonaventura Marguerite, Wald Abigail, Nikiforova Marina (2017). Breast Tumor Resembling Tall Cell Variant of Papillary Thyroid Carcinoma: A Solid Papillary Neoplasm With Characteristic Immunohistochemical Profile and Few Recurrent Mutations. Am J Clin Pathol.

[ref-4] Chiang Sarah, Weigelt Britta, Wen Huei-Chi, Pareja Fresia, Raghavendra Ashwini, Martelotto Luciano G., Burke Kathleen A., Basili Thais, Li Anqi, Geyer Felipe C., Piscuoglio Salvatore, Ng Charlotte K. Y., Jungbluth Achim A., Balss Jörg, Pusch Stefan, Baker Gabrielle M., Cole Kimberly S., Deimling Andreas, Batten Julie M., Marotti Jonathan D., Soh Hwei-Choo, McCalip Benjamin L., Serrano Jonathan, Lim Raymond S., Siziopikou Kalliopi P., Lu Song, Liu Xiaolong, Hammour Tarek, Brogi Edi, Snuderl Matija, Iafrate A. John, Reis-Filho Jorge S., Schnitt Stuart J. (2016). IDH2 Mutations Define a Unique Subtype of Breast Cancer with Altered Nuclear Polarity. Cancer Res.

[ref-5] Cameselle-Teijeiro J., Abdulkader I., Barreiro-Morandeira F., Ruiz-Ponte C., Reyes-Santías R., Chavez E., Sobrinho-Simões M. (2006). Breast tumor resembling the tall cell variant of papillary thyroid carcinoma: a case report. Int J Surg Pathol.

[ref-6] Yang WT, Bu H, Foschini MP, Schnitt SJ, Allison KH, Brogi E, Ellis IO, Fox SB, Morris EA, Sahin A (2019). Tall cell carcinoma with reversed polarity. Breast Tumours: WHO Classification of Tumours.

[ref-7] Alsadoun Nadjla, MacGrogan Gaëtan, Truntzer Caroline, Lacroix-Triki Magali, Bedgedjian Isabelle, Koeb Marie-Hélène, El Alam Elsy, Medioni Dan, Parent Michel, Wuithier Pascal, Robert Isabelle, Boidot Romain, Arnould Laurent (2018). Solid papillary carcinoma with reverse polarity of the breast harbors specific morphologic, immunohistochemical and molecular profile in comparison with other benign or malignant papillary lesions of the breast: a comparative study of 9 additional cases. Mod Pathol.

[ref-8] Foschini Maria P., Asioli Sofia, Foreid Susan, Cserni Gabor, Ellis Ian O., Eusebi Vincenzo, Rosai Juan (2017). Solid Papillary Breast Carcinomas Resembling the Tall Cell Variant of Papillary Thyroid Neoplasms: A Unique Invasive Tumor With Indolent Behavior. Am J Surg Pathol.

[ref-9] Toss Michael S., Billingham Kim, Egbuniwe Isioma U., Moreno Filipa, Abass Areeg, Rakha Emad A. (2019). Breast Tumours Resembling the Tall Cell Variant of Thyroid Papillary Carcinoma: Are They Part of the Papillary Carcinoma Spectrum or a Distinct Entity?. Pathobiology.

[ref-10] Pitino A., Squillaci S., Spairani C., Rassu P. C., Cosimi M. F. (2017). Tall cell variant of papillary breast carcinoma: an additional case with review of the literature. Pathologica.

[ref-11] Shaoxian Tang, Baohua Yu, Xiaoli Xu, Yufan Cheng, Xiaoyu Tu, Hongfen Lu, Rui Bi, Xiangjie Sun, Ruohong Shui, Wentao Yang (2017). Characterisation of GATA3 expression in invasive breast cancer: differences in histological subtypes and immunohistochemically defined molecular subtypes. J Clin Pathol.

[ref-12] Darb-Esfahani Silvia, Minckwitz Gunter, Denkert Carsten, Ataseven Beyhan, Högel Bernhard, Mehta Keyur, Kaltenecker Gabriele, Rüdiger Thomas, Pfitzner Berit, Kittel Kornelia, Fiedler Bettina, Baumann Klaus, Moll Roland, Dietel Manfred, Eidtmann Holger, Thomssen Christoph, Loibl Sibylle (2014). Gross cystic disease fluid protein 15 (GCDFP-15) expression in breast cancer subtypes. BMC Cancer.

[ref-13] Cohen Adam L., Holmen Sheri L., Colman Howard (2013). IDH1 and IDH2 mutations in gliomas. Curr Neurol Neurosci Rep.

[ref-14] Green Claire L., Evans Catherine M., Zhao Lu, Hills Robert K., Burnett Alan K., Linch David C., Gale Rosemary E. (2011). The prognostic significance of IDH2 mutations in AML depends on the location of the mutation. Blood.

[ref-15] Borger Darrell R., Tanabe Kenneth K., Fan Kenneth C., Lopez Hector U., Fantin Valeria R., Straley Kimberly S., Schenkein David P., Hezel Aram F., Ancukiewicz Marek, Liebman Hannah M., Kwak Eunice L., Clark Jeffrey W., Ryan David P., Deshpande Vikram, Dias-Santagata Dora, Ellisen Leif W., Zhu Andrew X., Iafrate A. John (2012). Frequent mutation of isocitrate dehydrogenase (IDH)1 and IDH2 in cholangiocarcinoma identified through broad-based tumor genotyping. Oncologist.

[ref-16] Whitehall V. L. J., Dumenil T. D., McKeone D. M., Bond C. E., Bettington M. L., Buttenshaw R. L., Bowdler L., Montgomery G. W., Wockner L. F., Leggett B. A. (2014). Isocitrate dehydrogenase 1 R132C mutation occurs exclusively in microsatellite stable colorectal cancers with the CpG island methylator phenotype. Epigenetics.

[ref-17] Amary M. Fernanda, Bacsi Krisztian, Maggiani Francesca, Damato Stephen, Halai Dina, Berisha Fitim, Pollock Robin, O'Donnell Paul, Grigoriadis Anita, Diss Tim, Eskandarpour Malihe, Presneau Nadège, Hogendoorn Pancras Cw, Futreal Andrew, Tirabosco Roberto, Flanagan Adrienne M. (2011). IDH1 and IDH2 mutations are frequent events in central chondrosarcoma and central and periosteal chondromas but not in other mesenchymal tumours. J Pathol.

[ref-18] Pareja Fresia, Silva Edaise M., Frosina Denise, Geyer Felipe C., Lozada John R., Basili Thais, Da Cruz Paula Arnaud, Zhong Elaine, Derakhshan Fatemeh, D'Alfonso Timothy, Wen Hannah Y., Giri Dilip D., Hayes Malcolm M., Krings Gregor, Bhargava Rohit, Palazzo Juan P., Rakha Emad A., Hoda Syed A., Sanders Melinda E., Collins Laura C., Schnitt Stuart J., Chen Yunn-Yi, Weigelt Britta, Jungbluth Achim A., Reis-Filho Jorge S., Brogi Edi (2020). Immunohistochemical analysis of IDH2 R172 hotspot mutations in breast papillary neoplasms: applications in the diagnosis of tall cell carcinoma with reverse polarity. Mod Pathol.

[ref-19] Basolo Fulvio, Giannini Riccardo, Monaco Carmen, Melillo Rosa Marina, Carlomagno Francesca, Pancrazi Martina, Salvatore Giuliana, Chiappetta Gennaro, Pacini Furio, Elisei Rossella, Miccoli Paolo, Pinchera Aldo, Fusco Alfredo, Santoro Massimo (2002). Potent mitogenicity of the RET/PTC3 oncogene correlates with its prevalence in tall-cell variant of papillary thyroid carcinoma. Am J Pathol.

[ref-20] Czarniecka Agnieszka, Oczko-Wojciechowska Małgorzata, Barczyński Marcin (2016). BRAF V600E mutation in prognostication of papillary thyroid cancer (PTC) recurrence. Gland Surg.

